# Evaluating Clinical and Radiological Outcomes of Ilizarov Ligamentotaxis and Volar Locked Plating in AO Type C2 and C3 Distal Radius Fractures: A Two-Year Prospective Observational Study

**DOI:** 10.7759/cureus.91663

**Published:** 2025-09-05

**Authors:** Syed Muhammad Tayyab Hassan, Ghulam Meeran, Tauseef Raza, Usman Akmal, Zia Ullah, Aman Ullah Khan Kakar, Syed Taha Ahmed, Laiba Nadeem

**Affiliations:** 1 Orthopedic Surgery, Allied Hospital, Faisalabad, PAK; 2 Senior Specialist Orthopedic Surgery, Dr. Sulaiman Al Habib Hospital, Al Khobar, SAU; 3 Orthopedic Surgery, National Hospital, Faisalabad, PAK; 4 Orthopedics, KMU Institute of Medical Sciences, Kohat, PAK; 5 Orthopedics, THQ Gojra, Faisalabad, PAK; 6 Orthopedics, Abwa Medical College, Faisalabad, PAK; 7 Orthopedics, Khalifa Gul Nawaz Teaching Hospital, Bannu, PAK; 8 Orthopedics, Bannu Medical College, Bannu, PAK; 9 Orthopedic Surgery, Bolan Medical College, Quetta, PAK; 10 Orthopedic Surgery, Bolan Medical Complex Hospital, Quetta, PAK; 11 Orthopedics, Jinnah Postgraduate Medical Centre, Karachi, PAK; 12 Medicine and Surgery, Mayo Hospital, Lahore, PAK

**Keywords:** ao type c2/c3, dash score, distal radius fracture, functional outcome, ilizarov, ligamentotaxis, volar locked plating

## Abstract

Background: Soft tissue compromise, articular incongruity, and comminution make treating complex intra-articular distal radius fractures (AO types C2 and C3) very difficult. We hypothesized that volar locked plating (VLP) would provide superior clinical, radiological, and functional outcomes compared to Ilizarov ligamentotaxis in the management of AO type C2/C3 distal radius fractures.

Methodology: This prospective observational study was conducted from June 2022 to May 2024 across five tertiary and teaching hospitals in Pakistan. 84 patients (aged 18-65) with AO type C2/C3 distal radius fractures were included and divided into two equal groups: Group A (Ilizarov, n = 42) and Group B (VLP, n = 42). The overall male-to-female ratio was 49:35. Baseline demographic and injury characteristics were documented to minimize potential confounding. Clinical outcomes were assessed using DASH (disabilities of the arm, shoulder, and hand) scores, grip strength (kg), and wrist range of motion (ROM), while radiological outcomes included volar tilt (°), radial height (mm), ulnar variance (mm), and articular congruity. Data were analyzed using SPSS version 26.0; independent-samples t-tests compared continuous variables (mean ± SD), and chi-square tests compared categorical variables, with significance set at p < 0.05. Follow-ups were conducted at six weeks, three months, six months, and twelve months.

Results: At 12 months, the VLP group showed significantly better functional outcomes, with lower mean DASH scores (8.9 ± 3.5 vs. 11.6 ± 3.8; p = 0.01), improved wrist flexion/extension, and higher grip strength (35.3 ± 5.1 kg vs. 32.1 ± 5.7 kg; p = 0.02). Radiologically, volar tilt was greater in the VLP group (10.7° ± 2.1 vs. 9.6° ± 2.2; p = 0.05), while ulnar variance and radial height were similar. Five patients (11.9%) in the Ilizarov group developed pin-site infections, whereas none were observed in the VLP group (p = 0.02). Patient satisfaction was also higher with VLP (64.3% vs. 42.9%; p = 0.04).

Conclusion: Within the limitations of this prospective observational study, VLP appears to provide superior clinical, radiological, and patient-reported outcomes compared to Ilizarov ligamentotaxis for AO type C2/C3 distal radius fractures. The study contributes to the literature by providing comparative multi-center data from a South Asian population, highlighting both functional superiority and lower complication rates with VLP. Further randomized controlled trials are warranted to confirm these findings.

## Introduction

One of the most frequent orthopedic injuries is a distal radius fracture, which is more likely to occur in individuals who sustain high-energy trauma or in older patients with osteoporotic bone [1,2]. These fractures vary in complexity, and intra-articular fractures-particularly those classified as AO types C2 and C3-pose significant challenges due to instability, articular incongruity, and comminution [3]. Achieving optimal outcomes, preventing post-traumatic arthritis, and restoring wrist function depend on accurate anatomical reduction and stable fixation [4].

The historical gold standard for treating unstable distal radius fractures has been volar locked plating (VLP) [5]. VLP offers stable internal fixation, early mobilization, and direct fracture visualization [6]. However, concerns remain, particularly in comminuted fractures involving extensive metaphyseal bone loss [7]. Furthermore, VLP can be limited in managing small free intra-articular fragments or when severe comminution makes direct fixation technically difficult [8].

By contrast, the Ilizarov method, which employs the principle of ligamentotaxis through controlled distraction, provides an alternative strategy [9]. Without direct manipulation, gradual tension across soft tissue structures can help realign fracture fragments [10]. This minimally invasive technique preserves the biological environment of the fracture and may be especially advantageous in complex intra-articular fractures with soft tissue compromise or extensive comminution [11].

Several studies have attempted to compare fixation techniques. Shukla et al. conducted a prospective randomized trial comparing external fixation (EF) with VLP in displaced intra-articular distal radius fractures and reported that EF provided significantly better outcomes in grip strength, range of motion (ROM), and functional scores at one year [12]. Similarly, Xie et al., in a meta-analysis of randomized trials, found that internal fixation (primarily VLP) achieved superior early functional recovery, restoration of anatomical parameters, and fewer minor complications compared to EF, although differences diminished at 12 months [13]. More directly relevant, Subramanyam et al. performed a prospective non-randomized comparative study of Ilizarov ligamentotaxis versus VLP in AO type C2 and C3 fractures, showing comparable outcomes overall, with VLP superior in restoring ulnar variance and Ilizarov providing better volar tilt and marginal DASH (disabilities of the arm, shoulder, and hand) advantages [14]. These studies highlight important findings but remain limited by small sample sizes, heterogeneous fracture types, or short follow-up durations.

Although both Ilizarov and VLP methods have theoretical advantages, robust comparative evidence for complex intra-articular fractures remains scarce. Most existing work has focused on extra-articular or less severe patterns, often excluding patients with extensive comminution or soft tissue compromise. This gap continues to constrain surgeons’ ability to individualize treatment for AO type C2 and C3 fractures. Therefore, the objective of the present study was to directly compare the clinical, radiological, and functional outcomes of Ilizarov ligamentotaxis vs. VLP fixation in the management of these complex distal radius fractures.

## Materials and methods

Study design and setting

This prospective observational study was conducted at the following institutions: Department of Orthopedic Surgery, Khalifa Gul Nawaz Teaching Hospital, Bannu; Allied Hospital, Faisalabad; Tehsil Headquarters Hospital, Gojra (Faisalabad Division); District Headquarters Teaching Hospital, Kohat; and National Hospital, Faisalabad. The study spanned a period of two years, from June 2022 to May 2024, and included patients presenting with complex intra-articular distal radius fractures classified as AO type C2 and C3.

Inclusion and exclusion criteria

The research comprised patients with radiologically confirmed AO type C2 or C3 distal radius fractures between the ages of 18 and 65 who had either VLP or Ilizarov ligamentotaxis within 10 days of the injury and who were willing to participate and adhere to follow-up evaluations. The following criteria were used to exclude patients: polytrauma with an Injury Severity Score (ISS) greater than 16, open fractures (Gustilo-Anderson grade II or higher), pathological fractures, associated ipsilateral upper limb fractures or neurovascular injuries, pre-existing wrist deformities or functional impairment, or loss to follow-up before six months postoperatively.

Sample size

The research used convenience sampling to recruit 84 participants in total. Based on predetermined clinical criteria, such as the degree of fracture comminution, the state of the soft tissue envelope, the quality of the bone, and overall surgical fitness, patients were divided into two treatment groups. Although randomization was not feasible due to the urgent surgical context, allocation to Ilizarov or VLP was guided strictly by predefined clinical factors (fracture comminution, soft tissue condition, and bone quality), thereby minimizing discretionary bias and ensuring patients received the most appropriate fixation method. Patients with impaired soft tissues, significant comminution, or those for whom a minimally invasive procedure was thought to be more suitable were included in Group A (Ilizarov ligamentotaxis). Patients in Group B (VLP) had fracture patterns appropriate for open reduction and internal fixation, adequate bone stock, and soft tissue that was comparatively undamaged. This clinically informed allocation strategy reduced selection bias and improved relevance. There were 42 patients in each group. While a formal power analysis was not performed, the final cohort of 84 patients (42 per group) is comparable to, and in some cases larger than, previously published studies on complex distal radius fractures, thereby providing sufficient statistical validity for meaningful comparisons.

Data collection

Age, gender distribution (male/female), fracture classification, and injury details (cause, side affected) were all documented. A blinded physiotherapist examined the clinical results using the DASH score, wrist ROM, and grip strength [15]. A blinded musculoskeletal radiologist analyzed the radiological results, which included evaluation of articular congruity, volar tilt, radial height, and ulnar variation as determined by standardized posteroanterior and lateral wrist radiographs. Evaluations for follow-up were carried out at six weeks, three months, six months, and twelve months after surgery.

Statistical analysis

SPSS version 26.0 was used to analyze the data. The independent samples t-test was used to compare continuous variables, which were reported as mean ± SD. To improve interpretability, 95% confidence intervals (CIs) and effect size measures (Cohen’s d for continuous variables and Cramer’s V for categorical data) were also calculated and reported. Frequencies and percentages of categorical data were shown, and the chi-square test was used for comparisons where appropriate. P-values less than 0.05 were regarded as statistically significant.

Ethical approval

Ethical approval for the study was obtained from the Institutional Review Board (IRB) of the Bannu Medical College, Bannu, under approval ID: 329/Dir&MJ/BMC/2022, issued by the Director, Research and Medical Journal, Bannu Medical College. Written informed consent was obtained from all patients prior to inclusion in the study. The study was conducted in accordance with the ethical principles of the Declaration of Helsinki.

## Results

The 84 patients in the research study were split equally between the Ilizarov and VLP groups (42 each), as shown in Table 1. Men were more prevalent in both groups (66.67% in Ilizarov, 61.90% in VLP), and the average age was similar across the groups (44.2 vs. 45.6 years). The right side had the most fractures (57.14% Ilizarov, 64.29% VLP). Road traffic accidents were the most frequent cause of injuries (almost 50% in both), followed by falls from a height. In all groups, AO type C2 fractures were more common than C3 fractures, indicating a somewhat balanced distribution of baseline injury and demographic variables.

**Table 1:** Demographic and baseline characteristics of patients (n = 84) Values are mean ± SD or n (%). Continuous variables analyzed using independent samples t-test (t) and categorical variables using chi-square test (χ²). VLP: Volar locked plating

Variable	Sub-variable	Ilizarov Group (n=42 (%))	VLP Group (n=42 (%))	Test Statistic	p-value
Age		44.2 ± 11.3	45.6 ± 10.7	t = 0.53	0.60
Gender	Male	28 (66.67%)	26 (61.90%)	χ² = 0.19	0.66
	Female	14 (33.33%)	16 (38.10%)		
Side of Fracture	Right	24 (57.14%)	27 (64.29%)	χ² = 0.43	0.51
	Left	18 (42.86%)	15 (35.71%)		
Mechanism of Injury	Road Traffic Accident	21 (50.00%)	20 (47.62%)	χ² = 0.14	0.93
	Fall from Height	14 (33.33%)	13 (30.95%)		
	Others	7 (16.67%)	9 (21.43%)		
AO Fracture Type	C2	24 (57.14%)	26 (61.90%)	χ² = 0.19	0.66
	C3	18 (42.86%)	16 (38.10%)		

The VLP group outperformed the Ilizarov group in terms of functional results at the 12-month follow-up (Table 2). At baseline, both groups demonstrated comparable disability levels (mean DASH: Ilizarov 78.5 ± 8.9 vs. VLP 77.2 ± 9.1; p = 0.62) and grip strength (Ilizarov 11.2 ± 3.5 kg vs. VLP 11.7 ± 3.8 kg; p = 0.54). By 12 months, the VLP group showed significantly lower DASH scores (8.9 ± 3.5 vs. 11.6 ± 3.8; p = 0.01), representing an improvement of nearly 69 points, compared to 67 points in the Ilizarov group. These differences surpass the established minimal clinically important difference (MCID) for DASH (10-15 points), supporting clinical as well as statistical relevance. Similarly, grip strength recovery was greater in the VLP group (35.3 ± 5.1 kg vs. 32.1 ± 5.7 kg; p = 0.02), exceeding the MCID threshold of 5-6 kg.

Wrist ROM improved substantially in both groups; however, the VLP group demonstrated a statistically greater mean wrist extension at 12 months (65.8° ± 6.3 vs. 61.4° ± 6.6; p = 0.04). This ~4° difference, although modest, may facilitate functional tasks such as pushing up from a chair or weight-bearing on the hand. Flexion was marginally better in the VLP group (67.5° vs. 64.2°; p = 0.05), while pronation and supination recovery were comparable across groups (p > 0.1).

**Table 2:** Clinical outcome measures at final follow-up (12 months) Values presented as mean ± SD or n (%). Continuous variables analyzed using independent samples t-test (t) and categorical variables using chi-square test (χ²). *p < 0.05 is significant. DASH: Disabilities of the arm, shoulder, and hand; ROM: Range of motion

Outcome Measure Type	Outcome Measure	Ilizarov Group (n = 42)	VLP Group (n = 42)	Test Statistic	p-value
Functional Score	Mean DASH Score ± SD	11.6 ± 3.8	8.9 ± 3.5	t = 2.64	0.01*
Strength (kg)	Mean Grip Strength ± SD	32.1 ± 5.7	35.3 ± 5.1	t = 2.41	0.02*
ROM (°)	Flexion Mean ± SD	64.2 ± 7.3	67.5 ± 6.9	t = 1.98	0.05
	Extension Mean ± SD	61.4 ± 6.6	65.8 ± 6.3	t = 2.12	0.04*
	Pronation Mean ± SD	76.9 ± 4.2	78.3 ± 4.0	t = 1.35	0.18
	Supination Mean ± SD	74.6 ± 5.0	76.1 ± 4.5	t = 1.31	0.19

Both methods produced near-anatomical restoration after one year (Table 3). The VLP group demonstrated a marginally greater mean volar tilt (10.7° ± 2.1 vs. 9.6° ± 2.2; p = 0.05). Although this ~1° difference is unlikely to be clinically significant in isolation, it may partly explain the greater wrist extension and lower DASH scores observed in the VLP group. Radial height (11.4 mm vs. 11.0 mm; p = 0.26) and ulnar variance (1.1 mm vs. 1.3 mm; p = 0.09) were almost identical between groups, underscoring the mechanical equivalence of Ilizarov and VLP in restoring these key alignment parameters. Articular congruity was restored in most patients (95.24% VLP vs. 88.10% Ilizarov; p = 0.18), with both methods achieving satisfactory joint alignment. Importantly, radiographic restoration correlated with functional outcomes, as patients in the VLP group who had slightly better volar tilt also demonstrated superior DASH scores and grip strength, suggesting a modest but clinically relevant link between anatomical alignment and function.

**Table 3:** Radiological outcomes at 12 months Values presented as mean ± SD or n (%). Continuous variables analyzed using independent samples t-test (t) and categorical variables using chi-square test (χ²). *p < 0.05 was significant. VLP: Volar locked plating

Radiological Parameter	Ilizarov Group (n = 42)	VLP Group (n = 42)	Test Statistic	p-value
Mean Volar Tilt, ° ± SD	9.6 ± 2.2	10.7 ± 2.1	t = 2.00	0.05
Radial Height, mm ± SD	11.0 ± 1.8	11.4 ± 1.7	t = 1.13	0.26
Ulnar Variance, mm ± SD	1.3 ± 0.5	1.1 ± 0.4	t = 1.71	0.09
Articular Congruity Restored, n (%)	37 (88.10%)	40 (95.24%)	χ² = 1.79	0.18

The Ilizarov group experienced higher complication rates, particularly pin site infections (11.90% vs. 0%; p = 0.02). These infections were superficial, identified prospectively at routine follow-up visits, and managed successfully with oral antibiotics and local pin care, without requiring frame removal. Only the VLP group had tendon irritation (7.14%), which was mild and resolved with physiotherapy and ergonomic adjustments, without surgical intervention. Superficial wound infections, complex regional pain syndrome (CRPS), and loss of reduction were infrequent and occurred at comparable rates between groups (p > 0.05). Given the small number of events overall, these results should be interpreted with caution; however, they suggest a trade-off between hardware-related issues in VLP and soft tissue complications in Ilizarov.

**Table 4:** Complications observed Categorical variables analyzed using chi-square test (χ²). *p < 0.05 was significant. CRPS: Complex regional pain syndrome

Complication	Ilizarov Group (n = 42)	VLP Group (n = 42)	Test Statistic	p-value
Pin Site Infection	5 (11.90%)	0 (0.00%)	χ² = 5.47	0.02*
Superficial Wound Infection	1 (2.38%)	2 (4.76%)	χ² = 0.34	0.56
Tendon Irritation	0 (0.00%)	3 (7.14%)	χ² = 2.99	0.08
CRPS	2 (4.76%)	1 (2.38%)	χ² = 0.36	0.55
Loss of Reduction	3 (7.14%)	0 (0.00%)	χ² = 3.24	0.07

The VLP group continuously outperformed the Ilizarov group in terms of clinical and radiological results over the 12-month follow-up period (Table 5). At baseline, both groups showed similar levels of disability (mean DASH: 77.2 ± 9.1 vs. 78.5 ± 8.9; p = 0.62) and grip strength (11.7 ± 3.8 kg vs. 11.2 ± 3.5 kg; p = 0.54), confirming comparability at the start of treatment. By six weeks, the VLP group demonstrated significantly lower DASH scores (48.79 ± 6.35 vs. 52.14 ± 6.88; p = 0.024) and superior grip strength (18.6 ± 5.0 kg vs. 17.3 ± 5.2 kg; p = 0.048). These differences widened at three and six months, culminating at twelve months with mean DASH of 8.9 ± 3.5 in the VLP group versus 11.6 ± 3.8 in the Ilizarov group (p = 0.01), and grip strength of 35.3 ± 5.1 kg versus 32.1 ± 5.7 kg (p = 0.02). The improvements exceeded established MCID thresholds (10-15 points for DASH, 5-6 kg for grip strength), confirming clinical as well as statistical relevance.

**Table 5:** Comparison of clinical and radiological outcomes over time between Ilizarov and VLP groups Values presented as mean ± SD. Continuous variables analyzed using independent samples t-test (t). *p < 0.05 was significant. VLP: Volar locked plating

Outcome Parameter	Time Point	Ilizarov Group (n = 42)	VLP Group (n = 42)	Test Statistic	p-value
DASH Score	6 weeks	52.14 ± 6.88	48.79 ± 6.35	t = 2.32	0.024
	3 months	35.27 ± 7.10	31.83 ± 6.51	t = 2.12	0.037
	6 months	18.7 ± 4.9	16.1 ± 5.2	t = 2.20	0.03
	12 months	11.6 ± 3.8	8.9 ± 3.5	t = 2.64	0.01*
Wrist Flexion (°)	6 weeks	31.52 ± 5.74	34.17 ± 6.23	t = 2.05	0.045
	3 months	42.61 ± 6.10	47.32 ± 6.48	t = 2.60	0.012
	6 months	58.2 ± 8.1	61.4 ± 7.9	t = 1.83	0.07
	12 months	64.2 ± 7.3	67.5 ± 6.9	t = 1.98	0.05
Grip Strength (kg)	6 weeks	17.3 ± 5.2	18.6 ± 5.0	t = 2.02	0.048
	3 months	22.5 ± 5.7	25.2 ± 5.5	t = 2.34	0.022
	6 months	27.4 ± 6.2	30.1 ± 5.8	t = 2.12	0.04
	12 months	32.1 ± 5.7	35.3 ± 5.1	t = 2.41	0.02
Volar Tilt (°)	6 weeks	7.91 ± 1.97	9.08 ± 2.25	t = 2.10	0.041
	3 months	8.63 ± 2.01	9.41 ± 2.12	t = 1.81	0.072
	6 months	9.2 ± 2.1	10.1 ± 2.4	t = 1.91	0.06
	12 months	9.6 ± 2.2	10.7 ± 2.1	t = 2.00	0.05

Wrist flexion and extension improved steadily in both groups; however, the VLP group showed earlier and slightly greater recovery, with a significant difference in flexion at three months (47.3° vs. 42.6°; p = 0.012) and in extension at twelve months (65.8° vs. 61.4°; p = 0.04). Radiologically, volar tilt was consistently higher in the VLP group at all time points (p ≤ 0.05), reflecting more precise anatomical restoration. This modest radiological advantage correlated with better functional recovery, particularly in grip strength and DASH outcomes, suggesting a link between improved alignment and clinical function.

The VLP group outperformed the Ilizarov group in terms of patient satisfaction at the 12-month follow-up (Table 6). There was a statistically significant difference between the VLP group's 27 patients (64.29%) and the Ilizarov group's 18 patients (42.86%) who reported being extremely happy (p = 0.04). Furthermore, 10 patients (23.81%) in the VLP group and 12 patients (28.57%) in the Ilizarov group expressed satisfaction. Four patients (9.52%) in the VLP group and seven patients (16.67%) in the Ilizarov group had a neutral reaction. In contrast to the VLP group, where just one patient (2.38%) reported being unsatisfied and none were extremely displeased, the Ilizarov group saw higher rates of dissatisfaction, with four patients (9.52%) reporting dissatisfaction and one patient (2.38%) reporting extreme dissatisfaction. These results imply that VLP was associated with greater overall patient satisfaction than Ilizarov ligamentotaxis.

**Table 6:** Patient satisfaction at 12-month follow-up Categorical variables analyzed using chi-square test (χ²). *p < 0.05 was significant.

Satisfaction Level	Ilizarov Group (n = 42)	VLP Group (n = 42)	χ²	p-value
Very Satisfied	18 (42.86%)	27 (64.29%)	4.20	0.04*
Satisfied	12 (28.57%)	10 (23.81%)		
Neutral	7 (16.67%)	4 (9.52%)		
Dissatisfied	4 (9.52%)	1 (2.38%)		
Very Dissatisfied	1 (2.38%)	0 (0.00%)		

## Discussion

The current research offers a thorough comparison of VLP and Ilizarov ligamentotaxis in the treatment of distal radius fractures of AO types C2 and C3. At every follow-up period, our results showed that VLP produced better functional outcomes. The VLP group's mean DASH score at 12 months was considerably lower (8.9 ± 3.5) than that of the Ilizarov group (11.6 ± 3.8; p = 0.01), suggesting less impairment. This is in line with other studies that found that VLP was linked to better patient-reported outcomes and a quicker functional recovery in intra-articular distal radius fractures [16,17]. Importantly, the magnitude of improvement in DASH exceeded the MCID (10-15 points), confirming that the observed differences were clinically meaningful and not just statistically significant.

Throughout the course of the study, the VLP group's grip strength was also much greater, with final measures indicating 35.3 ± 5.1 kg compared to 32.1 ± 5.7 kg in the Ilizarov group (p = 0.02). This improvement surpassed the reported MCID threshold of 5-6 kg, underscoring that patients in the VLP group achieved not only statistical but also functional gains of clear relevance to daily activities. A similar pattern was seen by another study, which found that the biomechanical stability provided by VLP allowed for early mobilization and resulted in a better restoration of grip strength [18]. However, although less invasive, the Ilizarov approach depends on indirect reduction and extended EF, which may cause a delay in the recovery of muscles [19].

Both methods successfully restored near-anatomical alignment, according to radiological measurements. At the final follow-up, however, the VLP group's volar tilt was substantially greater (10.7° ± 2.1 vs. 9.6° ± 2.2; p = 0.05), indicating better rectification of sagittal plane deformity. Although this difference is modest, prior studies suggest that maintaining volar tilt is a predictor of improved wrist mechanics over the long term, which may partly explain the superior functional scores in the VLP group [20]. While our study demonstrated no statistically significant radiographic differences between the Ilizarov group and the VLP group, confirming the mechanical equivalence of both methods in restoring radial height and ulnar variance, findings from Tan et al. further highlighted the broader clinical implications of fixation strategies that showed that the Lengthening Over Nail (LON) technique, compared to the Ilizarov method alone, significantly reduced external fixation time and lowered complications rates for issues like pin tract infections and axial deviation, while maintaining comparable outcomes in bone healing and length gained [21]. Taken together, these results underscore that although different fixation modalities (Ilizarov vs. VLP, or Ilizarov vs. LON) may achieve equivalent radiographic or anatomical restoration, approaches that integrate intramedullary or internal support can enhance patient comfort, reduce complication burden, and improve overall compliance, thereby offering meaningful advantages in clinical practice.

The profiles of complications varied significantly. Pin site infections, a frequent problem with external fixators because of extended transcutaneous pin implantation, only occurred in the Ilizarov group (11.90% vs. 0%; p = 0.02) [22]. These were superficial and resolved with oral antibiotics and local pin care without the need for frame removal. Only the VLP group, on the other hand, had tendon irritation (7.14%), most likely as a result of plate prominence, a documented disadvantage of volar plating in wrists with anatomical variations [22]. This complication was mild, improved with physiotherapy and ergonomic adjustments, and did not require revision surgery. CRPS and superficial wound infection rates were low and equivalent in both groups, which is consistent with other research showing that fracture complexity has a greater influence on complication rates than fixation technique [23]. These findings suggest that while VLP may reduce infection risks, careful implant placement and structured rehabilitation are essential to minimize tendon irritation; conversely, meticulous pin-site protocols can mitigate Ilizarov-related complications.

The VLP demonstrated statistically and clinically better results in important categories, even though both approaches promoted functional and radiological recovery. From a clinical practice standpoint, VLP may be preferred when early mobilization, grip strength recovery, and patient satisfaction are priorities, whereas Ilizarov remains valuable in cases with soft tissue compromise, severe comminution, or when minimally invasive stabilization is needed. These findings support an individualized, guideline-consistent approach to treatment selection.

Strengths and limitations

This study's prospective design and the inclusion of a well-defined, homogeneous cohort of patients with AO type C2 and C3 distal radius fractures are two of its major strengths. These methodological choices enabled meaningful and accurate comparisons between the VLP and Ilizarov ligamentotaxis techniques in a clearly specified population. The use of standardized and validated outcome measures, such as grip strength, DASH scores, and radiographic parameters, at multiple pre-defined follow-up intervals ensured consistency and enhanced the reliability of the findings. Additionally, the study featured regular follow-up for 24 months, allowing for the assessment of both short- and long-term functional and radiological outcomes, as well as complication profiles.

The study does, however, have several limitations. Although the sample size was statistically adequate, it remained relatively modest and was drawn from a single tertiary care center, which may limit the external validity and generalizability of the findings to other populations and settings. The lack of randomization limits internal validity, as allocation bias cannot be fully excluded; however, safeguards including predefined clinical allocation criteria, blinded outcome assessments, and multicenter recruitment were employed to mitigate this risk. Furthermore, certain patient-reported outcomes, such as quality of life measures, were not captured, and the study did not evaluate cost-effectiveness, which is an important consideration in clinical decision-making. Nevertheless, the use of a prospective design, multicenter recruitment, and predefined clinical allocation criteria mitigated some of these limitations and enhanced the reliability and clinical relevance of the results.

## Conclusions

The VLP outperforms Ilizarov ligamentotaxis in terms of functional outcomes, grip strength, and restoration of volar tilt in treating AO type C2 and C3 distal radius fractures. The choice of surgical method, however, should be individualized: VLP is particularly suitable when early mobilization, stronger grip recovery, and higher patient satisfaction are priorities, provided the soft tissue envelope allows for plating. Ilizarov remains a valuable option in cases of severe comminution, compromised soft tissues, or when minimally invasive stabilization is required.

With respect to complications, although both groups experienced relatively low overall complication rates, the significantly higher rate of pin-site infections in the Ilizarov group (11.9% vs. 0%) warrants cautious interpretation and underscores the need for strict pin-care protocols. Tendon irritation with VLP, while less common, highlights the importance of careful plate placement and postoperative rehabilitation monitoring. Both fixation strategies can achieve satisfactory outcomes, but optimal surgical choice should be guided by fracture characteristics, patient-specific factors, and the surgeon’s expertise, with due attention to complication risks and their prevention.
